# 2-Oxo-2*H*-chromen-7-yl 3-methyl­butano­ate

**DOI:** 10.1107/S2414314625001610

**Published:** 2025-02-28

**Authors:** Akoun Abou, Hypolite Bazié, Ludovic Akonan, Abdoulaye Djandé, Pierre Francotte

**Affiliations:** a Joint Research and Innovation Unit for Engineering Sciences and Techniques, (UMRI STI), Research Team: Instrumentation, Image and Spectroscopy, Félix Houphouet-Boigny National Polytechnic Institute, BP 1093 Yamoussoukro, Côte d’Ivoire; b Laboratory of Molecular Chemistry and Materials (LC2M), Research Team: Organic Chemistry and Phytochemistry, University Joseph KI-ZERBO, 03 BP 7021 Ouagadougou 03, Burkina Faso; c Laboratory of Fundamental and Applied Physics, Nangui Abrogoua University, Abidjan, Côte d’Ivoire; d Center for Interdisciplinary Research on Medicinal Chemistry, University of Liège, Avenue Hippocrate 15 (B36), B-4000, Liège, Belgium; Benemérita Universidad Autónoma de Puebla, México

**Keywords:** crystal structure, coumarin derivative, umbelliferone, hydrogen bonds

## Abstract

The title compound, C_14_H_14_O_4_, was synthesized by *O*-acyl­ation of umbelliferone with isovaleryl chloride in the presence of diethyl ether as a solvent and pyridine as a base. The side chain moiety *i.e.* the acetate fragment linking to the methyl­ethyl group is almost orthogonal to the almost planar (r.m.s deviation = 0.020 Å) coumarin ring system, making an angle of 76.26 (7)°. In the crystal, the mol­ecules form centrosymmetric dimers through pairwise C—H⋯O hydrogen bonds, generating *R*_2_^2^(8) and *R*_2_^2^(18) loops that lie within the crystallographic *ac* plane and propagate along the [001] direction.

## Abstract 2

In the crystal, mol­ecules form centrosymmetric hydrogen-bonded dimers through pairwise C—H⋯O inter­actions, generating *R*_2_^2^(8) and *R*_2_^2^(18) loops that lie within the crystallographic *ac* plane and propagate along the [001] direction.

## Graphical abstract

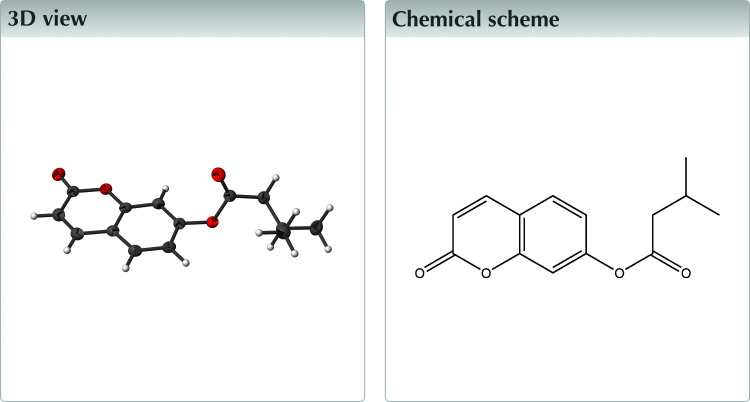

## Structure description

Mol­ecules containing the coumarin moiety have attracted the attention of researchers since examples of these compounds have been shown to have extensive biological properties, including anti-HIV (Yu *et al.*, 2003 ▸, 2007 ▸), anti-coagulant (Abernethy *et al.*, 1969 ▸), anti-oxidant (Vukovic *et al.*, 2010 ▸), anti-tumour (Basanagouda *et al.*, 2009 ▸), anti-bacterial (Vukovic *et al.*, 2010 ▸) and anti-inflammatory (Emmanuel-Giota *et al.*, 2001 ▸) activity. They are also used in the perfumery and agrochemical industries as activators and stabilizers (Bauer *et al.*, 1988 ▸; Boisde & Meuly, 1993 ▸).

**Figure 1:**
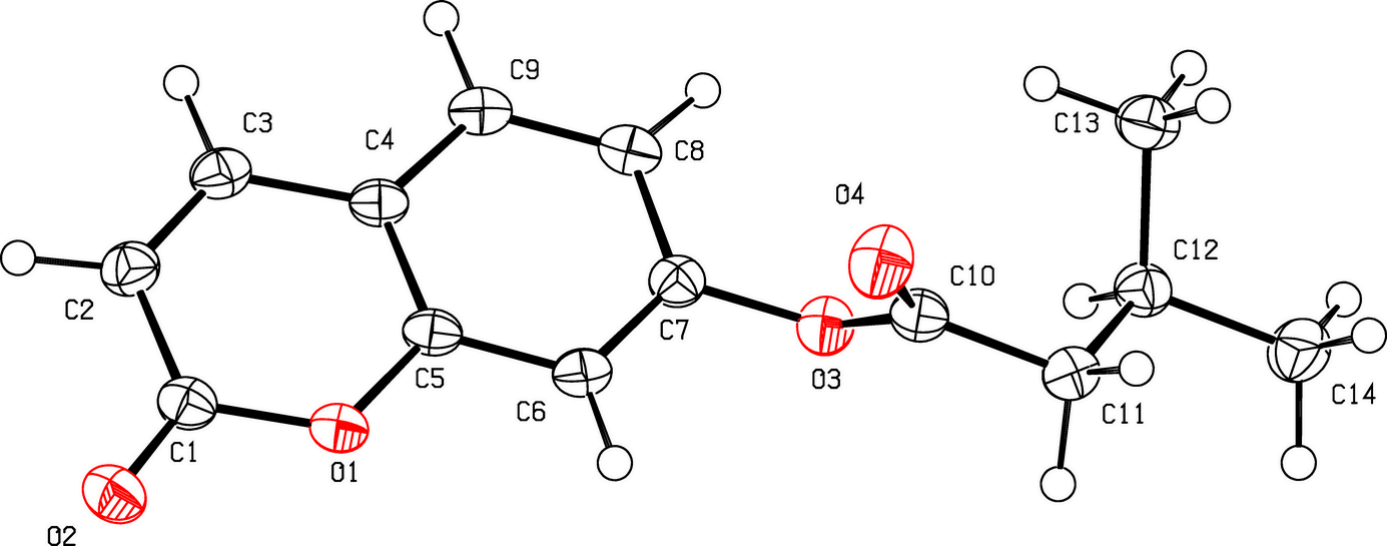
Mol­ecular structure of the title compound with the atomic numbering scheme. Displacement ellipsoids are drawn at the 50% probability level. H atoms are shown as spheres of arbitrary radius.

**Figure 2:**
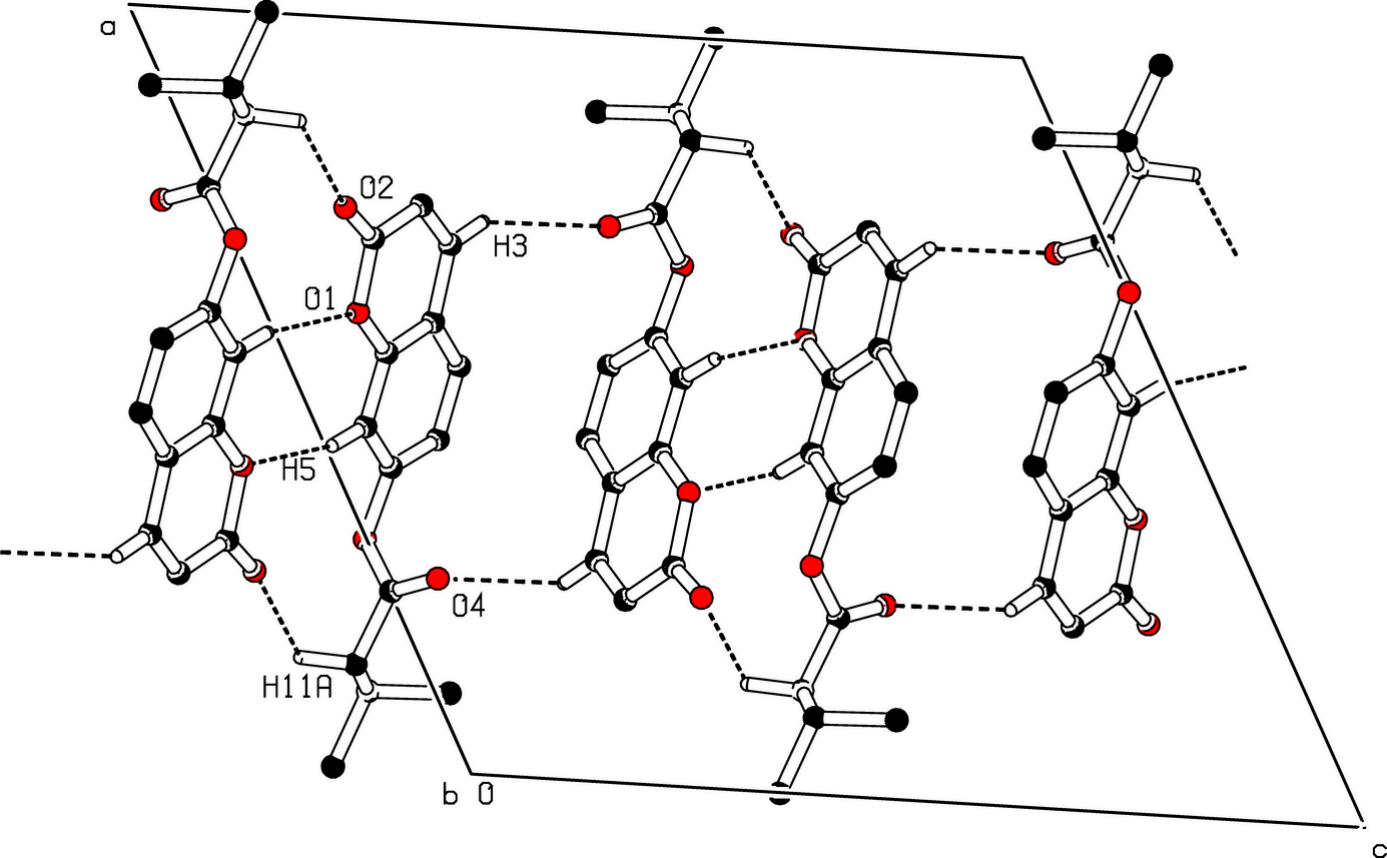
A view of the crystal packing showing the association of mol­ecules into centrosymmetric dimers through C—H⋯O hydrogen bonds, forming 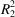
(8) and 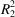
(18) loops extending parallel to the *ac* crystallographic plane. H atoms not involved in hydrogen bonding have been omitted for clarity.

IIn this work, we describe the synthesis and structure of the title compound, C_14_H_14_O_4_, Fig. 1 ▸. An analysis of the bond lengths in this structure shows a slightly uneven distribution around the pyrone ring, as indicated by the lengths of the C2=C3 [1.345 (3) Å] and C1—C2 [1.453 (3) Å] bonds, which are shorter and longer, respectively, than what would be expected for a C_ar_—C_ar_ bond. This suggests that the electron density is less concentrated in the C2=C3 bond of the pyran-2-one ring, resulting in the formation of a double bond, as observed in other coumarin ester derivatives (Abou *et al.*, 2020 ▸; Koulabiga *et al.*, 2024 ▸; Yao *et al.*, 2024 ▸). Furthermore, the structure highlights an almost planar coumarin ring system (puckering τ parameter = 0.7; Spek, 2009 ▸). Likewise, the crystal structure reveals the generation of dimeric units *via* C—H⋯O inter­actions. These dimers are linked by further C—H⋯O contacts into chains along the [001] direction (Table 1 ▸, Fig. 2 ▸).

## Synthesis and crystallization

To a solution of isovaleryl chloride (0.76 ml, 6.17 mmol, 1 equiv.) in dried diethyl ether (16 ml) was added dried pyridine (2.31 ml, 4.7 equiv.) and umbelliferone (1 g, 6.17 mmol, 1 equiv.) in small portions over 30 min, with vigorous stirring. The reaction mixture was left stirring at room temperature for 3 h. The resulting mixture was next poured into a separating funnel containing 40 ml of chloro­form and washed with diluted hydro­chloric acid solution until the pH was 2–3. The organic phase was extracted, washed with water to neutrality, dried with magnesium sulfate and the solvent removed *in vacuo*. The obtained crude product was filtered off with suction, washed with petroleum ether and recrystallized from the mixed solvents of chloro­form–hexane (1:3), yielding a white powder of the title compound, 2-oxo-2*H*-chromen-7-yl-3-methyl­butano­ate (0.92 g, 60%). Colourless crystals suitable for single-crystal X-ray diffraction analysis were then obtained from an acetone solution, after the solvent was allowed to evaporate slowly at ambient conditions.

## Refinement

Crystal data, data collection and structure refinement details are summarized in Table 2 ▸. Three reflections, (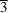
46), (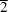
04), (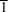
04) with Δ*F*/σ(*F*) higher than 10, were found to have too low intensities, caused by a systematic error, probably by shielding by the beam-stop inter­ference for reflections (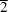
04), (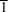
04) while for (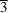
46) at higher 2θ angles, the less area irradiated would have an effect of decreasing diffraction intensity. The depth of penetration of the beam becomes commensurably deeper with higher angles. This effectively increases background as well as a sample displacement effect.. They were omitted from the refinement.

## Supplementary Material

Crystal structure: contains datablock(s) I. DOI: 10.1107/S2414314625001610/bh4093sup1.cif

Structure factors: contains datablock(s) I. DOI: 10.1107/S2414314625001610/bh4093Isup2.hkl

Supporting information file. DOI: 10.1107/S2414314625001610/bh4093Isup3.cml

CCDC reference: 2426608

Additional supporting information:  crystallographic information; 3D view; checkCIF report

## Figures and Tables

**Table 1:** Hydrogen-bond geometry (Å, °)

*D*—H⋯*A*	*D*—H	H⋯*A*	*D*⋯*A*	*D*—H⋯*A*
C3—H3⋯O4^i^	0.98 (3)	2.46 (3)	3.296 (2)	142.6 (19)
C6—H5⋯O1^ii^	0.96 (3)	2.51 (3)	3.444 (2)	167 (2)
C11—H11*A*⋯O2^ii^	1.03 (3)	2.60 (3)	3.245 (2)	120.5 (18)

Symmetry codes: (i) 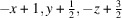; (ii) 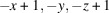.

**Table 2:** Experimental details

Crystal data	
Chemical formula	C_14_H_14_O_4_
*M*_r_	246.25
Crystal system, space group	Monoclinic, *P*2_1_/*c*
Temperature (K)	296
*a*, *b*, *c* (Å)	15.370 (3), 5.4488 (10), 16.339 (3)
β (°)	117.426 (7)
*V* (Å^3^)	1214.5 (4)
*Z*	4
Radiation type	Mo *K*α
μ (mm^−1^)	0.10
Crystal size (mm)	0.45 × 0.44 × 0.16

Data collection	
Diffractometer	SuperNova, Dual, Cu at home/near, AtlasS2
Absorption correction	Multi-scan (*CrysAlis PRO*; Rigaku OD, 2022 ▸)
*T*_min_, *T*_max_	0.956, 1.000
No. of measured, independent and observed [*I* > 2σ(*I*)] reflections	41227, 3741, 2799
*R*_int_	0.068
(sin θ/λ)_max_ (Å^−1^)	0.718

Refinement	
*R*[*F*^2^ > 2σ(*F*^2^)], *wR*(*F*^2^), *S*	0.066, 0.203, 1.03
No. of reflections	41227
No. of parameters	219
H-atom treatment	All H-atom parameters refined
Δρ_max_, Δρ_min_ (e Å^−3^)	0.50, −0.39

Computer programs: *CrysAlis PRO* (Rigaku OD, 2022 ▸), *SHELXT* (Sheldrick, 2015*a* ▸), *PLATON* (Spek, 2020 ▸), *SHELXL2018/3* (Sheldrick, 2015*b* ▸), *publCIF* (Westrip, 2010 ▸) and *WinGX* (Farrugia, 2012 ▸).

## full crystallographic data

### 2-Oxo-2H-chromen-7-yl 3-methylbutanoate Crystal data

**Table:**

C_14_H_14_O_4_	*F*(000) = 520
*M**_r_* = 246.25	*D*_x_ = 1.347 Mg m^−^^3^
Monoclinic, *P*2_1_/*c*	Melting point = 341–343 K
Hall symbol: -P 2ybc	Mo *K*α radiation, λ = 0.71073 Å
*a* = 15.370 (3) Å	Cell parameters from 3741 reflections
*b* = 5.4488 (10) Å	θ = 5.0–61.4°
*c* = 16.339 (3) Å	µ = 0.10 mm^−^^1^
β = 117.426 (7)°	*T* = 296 K
*V* = 1214.5 (4) Å^3^	Prism, white
*Z* = 4	0.45 × 0.44 × 0.16 mm

### 2-Oxo-2H-chromen-7-yl 3-methylbutanoate Data collection

**Table:**

SuperNova, Dual, Cu at home/near, AtlasS2 diffractometer	3741 independent reflections
Radiation source: micro-focus sealed X-ray tube	2799 reflections with *I* > 2σ(*I*)
Mirror monochromator	*R*_int_ = 0.068
Detector resolution: 5.3048 pixels mm^-1^	θ_max_ = 30.7°, θ_min_ = 2.5°
ω scans	*h* = −22→21
Absorption correction: multi-scan (CrysAlisPro; Rigaku OD, 2022)	*k* = −7→7
*T*_min_ = 0.956, *T*_max_ = 1.000	*l* = −23→23
41227 measured reflections	

### 2-Oxo-2H-chromen-7-yl 3-methylbutanoate Refinement

**Table:**

Refinement on *F*^2^	Primary atom site location: dual
Least-squares matrix: full	Secondary atom site location: difference Fourier map
*R*[*F*^2^ > 2σ(*F*^2^)] = 0.066	Hydrogen site location: difference Fourier map
*wR*(*F*^2^) = 0.203	All H-atom parameters refined
*S* = 1.03	*w* = 1/[σ^2^(*F*_o_^2^) + (0.1174*P*)^2^ + 0.7176*P*] where *P* = (*F*_o_^2^ + 2*F*_c_^2^)/3
41227 reflections	(Δ/σ)_max_ < 0.001
219 parameters	Δρ_max_ = 0.50 e Å^−^^3^
0 restraints	Δρ_min_ = −0.39 e Å^−^^3^

### 2-Oxo-2H-chromen-7-yl 3-methylbutanoate Special details

**Table:**

Refinement. All non-H atoms were refined anisotropically. H atoms were located in difference Fourier maps and refined freely with an isotropic displacement parameter.

### 2-Oxo-2H-chromen-7-yl 3-methylbutanoate Fractional atomic coordinates and isotropic or equivalent isotropic displacement parameters (Å^2^)

**Table:**

	*x*	*y*	*z*	*U*_iso_*/*U*_eq_	
O3	0.30502 (9)	0.5548 (2)	0.49786 (9)	0.0262 (3)	
O1	0.60720 (9)	0.1291 (2)	0.60615 (8)	0.0247 (3)	
O2	0.74608 (10)	−0.0712 (3)	0.64517 (10)	0.0315 (3)	
O4	0.25820 (10)	0.2488 (3)	0.56157 (10)	0.0362 (4)	
C5	0.55518 (12)	0.3187 (3)	0.61799 (11)	0.0225 (3)	
C4	0.60061 (12)	0.4940 (3)	0.68735 (11)	0.0230 (3)	
C7	0.40292 (12)	0.5249 (3)	0.56429 (11)	0.0236 (3)	
C6	0.45580 (13)	0.3301 (3)	0.55611 (11)	0.0240 (3)	
C3	0.70488 (13)	0.4686 (3)	0.74636 (12)	0.0268 (4)	
C11	0.13750 (13)	0.4489 (4)	0.42324 (12)	0.0276 (4)	
C1	0.70685 (13)	0.1025 (3)	0.66096 (12)	0.0249 (3)	
C12	0.10241 (13)	0.7130 (3)	0.42369 (12)	0.0262 (4)	
C10	0.23739 (13)	0.3983 (3)	0.50137 (12)	0.0257 (3)	
C9	0.54337 (13)	0.6854 (3)	0.69455 (12)	0.0260 (3)	
C2	0.75496 (13)	0.2830 (4)	0.73351 (12)	0.0279 (4)	
C8	0.44435 (13)	0.7026 (3)	0.63285 (12)	0.0258 (3)	
C14	−0.00084 (16)	0.7484 (4)	0.34503 (14)	0.0361 (4)	
C13	0.10591 (15)	0.7726 (4)	0.51641 (14)	0.0312 (4)	
H5	0.4273 (19)	0.210 (5)	0.5086 (19)	0.040 (7)*	
H3	0.7378 (17)	0.589 (5)	0.7957 (17)	0.036 (6)*	
H9	0.5763 (18)	0.811 (5)	0.7445 (19)	0.041 (7)*	
H8	0.4032 (18)	0.849 (5)	0.6331 (18)	0.037 (6)*	
H2	0.8282 (17)	0.258 (4)	0.7702 (16)	0.027 (6)*	
H11A	0.1421 (18)	0.418 (5)	0.3633 (18)	0.039 (7)*	
H14A	−0.051 (2)	0.631 (6)	0.352 (2)	0.048 (7)*	
H14B	−0.0221 (17)	0.929 (5)	0.3426 (17)	0.034 (6)*	
H11B	0.0901 (18)	0.329 (5)	0.4256 (17)	0.035 (6)*	
H12	0.1473 (16)	0.826 (4)	0.4176 (15)	0.026 (5)*	
H13A	0.0868 (17)	0.946 (5)	0.5179 (17)	0.036 (6)*	
H13B	0.1761 (18)	0.750 (4)	0.5683 (18)	0.033 (6)*	
H13C	0.059 (2)	0.656 (6)	0.5256 (19)	0.052 (8)*	
H14C	−0.005 (2)	0.709 (5)	0.282 (2)	0.051 (8)*	

### 2-Oxo-2H-chromen-7-yl 3-methylbutanoate Atomic displacement parameters (Å^2^)

**Table:**

	*U*^11^	*U*^22^	*U*^33^	*U*^12^	*U*^13^	*U*^23^
O3	0.0272 (6)	0.0259 (6)	0.0257 (6)	0.0001 (5)	0.0122 (5)	0.0045 (5)
O1	0.0292 (6)	0.0230 (6)	0.0239 (6)	0.0005 (5)	0.0141 (5)	−0.0023 (5)
O2	0.0351 (7)	0.0299 (7)	0.0336 (7)	0.0045 (5)	0.0193 (6)	−0.0002 (5)
O4	0.0338 (7)	0.0409 (8)	0.0323 (7)	−0.0023 (6)	0.0138 (6)	0.0128 (6)
C5	0.0300 (8)	0.0205 (7)	0.0202 (7)	0.0000 (6)	0.0144 (6)	0.0010 (6)
C4	0.0293 (8)	0.0228 (7)	0.0196 (7)	−0.0012 (6)	0.0136 (6)	0.0009 (6)
C7	0.0266 (8)	0.0250 (8)	0.0211 (7)	−0.0005 (6)	0.0126 (6)	0.0031 (6)
C6	0.0291 (8)	0.0233 (8)	0.0205 (7)	−0.0021 (6)	0.0123 (6)	−0.0010 (6)
C3	0.0309 (8)	0.0291 (8)	0.0207 (7)	−0.0023 (7)	0.0121 (6)	−0.0005 (6)
C11	0.0277 (8)	0.0298 (9)	0.0241 (8)	0.0000 (7)	0.0108 (6)	−0.0022 (7)
C1	0.0301 (8)	0.0245 (8)	0.0234 (7)	0.0009 (6)	0.0152 (6)	0.0034 (6)
C12	0.0278 (8)	0.0281 (8)	0.0246 (8)	0.0000 (6)	0.0138 (6)	0.0015 (6)
C10	0.0289 (8)	0.0259 (8)	0.0242 (8)	−0.0002 (6)	0.0139 (6)	0.0008 (6)
C9	0.0336 (8)	0.0239 (8)	0.0228 (7)	−0.0022 (6)	0.0150 (7)	−0.0033 (6)
C2	0.0287 (8)	0.0318 (9)	0.0224 (8)	−0.0005 (7)	0.0112 (6)	0.0009 (7)
C8	0.0341 (9)	0.0226 (8)	0.0254 (8)	0.0011 (6)	0.0177 (7)	0.0000 (6)
C14	0.0345 (10)	0.0391 (11)	0.0289 (9)	0.0071 (8)	0.0097 (8)	0.0023 (8)
C13	0.0348 (9)	0.0335 (10)	0.0294 (9)	−0.0013 (7)	0.0184 (8)	−0.0033 (7)

### 2-Oxo-2H-chromen-7-yl 3-methylbutanoate Geometric parameters (Å, º)

**Table:**

O3—C10	1.366 (2)	C11—H11A	1.03 (3)
O3—C7	1.402 (2)	C11—H11B	0.99 (3)
O1—C5	1.373 (2)	C1—C2	1.453 (3)
O1—C1	1.380 (2)	C12—C14	1.526 (3)
O2—C1	1.212 (2)	C12—C13	1.526 (3)
O4—C10	1.201 (2)	C12—H12	0.96 (2)
C5—C6	1.392 (2)	C9—C8	1.388 (3)
C5—C4	1.398 (2)	C9—H9	1.00 (3)
C4—C9	1.404 (2)	C2—H2	1.01 (2)
C4—C3	1.447 (2)	C8—H8	1.02 (3)
C7—C6	1.380 (2)	C14—H14A	1.04 (3)
C7—C8	1.393 (2)	C14—H14B	1.03 (3)
C6—H5	0.96 (3)	C14—H14C	1.03 (3)
C3—C2	1.345 (3)	C13—H13A	0.99 (3)
C3—H3	0.98 (3)	C13—H13B	1.03 (2)
C11—C10	1.502 (2)	C13—H13C	1.03 (3)
C11—C12	1.538 (3)		
			
C10—O3—C7	117.47 (13)	C13—C12—C11	110.59 (15)
C5—O1—C1	122.11 (13)	C14—C12—H12	110.5 (13)
O1—C5—C6	116.52 (14)	C13—C12—H12	105.5 (13)
O1—C5—C4	121.48 (15)	C11—C12—H12	109.0 (14)
C6—C5—C4	121.98 (15)	O4—C10—O3	122.46 (16)
C5—C4—C9	118.50 (16)	O4—C10—C11	127.06 (17)
C5—C4—C3	117.45 (15)	O3—C10—C11	110.44 (15)
C9—C4—C3	124.04 (16)	C8—C9—C4	120.59 (16)
C6—C7—C8	122.74 (16)	C8—C9—H9	121.2 (15)
C6—C7—O3	118.98 (15)	C4—C9—H9	118.2 (15)
C8—C7—O3	118.16 (15)	C3—C2—C1	121.56 (17)
C7—C6—C5	117.53 (15)	C3—C2—H2	124.7 (13)
C7—C6—H5	122.5 (15)	C1—C2—H2	113.7 (13)
C5—C6—H5	119.9 (15)	C9—C8—C7	118.63 (16)
C2—C3—C4	120.40 (16)	C9—C8—H8	121.7 (15)
C2—C3—H3	121.0 (14)	C7—C8—H8	119.5 (15)
C4—C3—H3	118.6 (14)	C12—C14—H14A	111.2 (16)
C10—C11—C12	113.02 (15)	C12—C14—H14B	109.9 (13)
C10—C11—H11A	106.7 (14)	H14A—C14—H14B	111 (2)
C12—C11—H11A	109.6 (15)	C12—C14—H14C	112.4 (16)
C10—C11—H11B	109.1 (14)	H14A—C14—H14C	106 (2)
C12—C11—H11B	110.5 (15)	H14B—C14—H14C	107 (2)
H11A—C11—H11B	108 (2)	C12—C13—H13A	110.6 (15)
O2—C1—O1	117.13 (16)	C12—C13—H13B	109.7 (14)
O2—C1—C2	125.87 (17)	H13A—C13—H13B	107.9 (19)
O1—C1—C2	116.99 (15)	C12—C13—H13C	108.1 (16)
C14—C12—C13	110.90 (16)	H13A—C13—H13C	110 (2)
C14—C12—C11	110.18 (15)	H13B—C13—H13C	110 (2)
			
C1—O1—C5—C6	177.72 (14)	C5—O1—C1—C2	0.9 (2)
C1—O1—C5—C4	−0.8 (2)	C10—C11—C12—C14	−177.74 (16)
O1—C5—C4—C9	178.90 (14)	C10—C11—C12—C13	−54.8 (2)
C6—C5—C4—C9	0.5 (2)	C7—O3—C10—O4	4.3 (3)
O1—C5—C4—C3	0.1 (2)	C7—O3—C10—C11	−177.61 (14)
C6—C5—C4—C3	−178.32 (15)	C12—C11—C10—O4	119.0 (2)
C10—O3—C7—C6	74.6 (2)	C12—C11—C10—O3	−58.91 (19)
C10—O3—C7—C8	−109.20 (18)	C5—C4—C9—C8	−1.4 (3)
C8—C7—C6—C5	−2.0 (3)	C3—C4—C9—C8	177.38 (16)
O3—C7—C6—C5	174.03 (14)	C4—C3—C2—C1	−0.2 (3)
O1—C5—C6—C7	−177.36 (14)	O2—C1—C2—C3	−179.25 (18)
C4—C5—C6—C7	1.1 (2)	O1—C1—C2—C3	−0.4 (3)
C5—C4—C3—C2	0.4 (2)	C4—C9—C8—C7	0.6 (3)
C9—C4—C3—C2	−178.36 (17)	C6—C7—C8—C9	1.2 (3)
C5—O1—C1—O2	179.85 (15)	O3—C7—C8—C9	−174.88 (14)

### 2-Oxo-2H-chromen-7-yl 3-methylbutanoate Hydrogen-bond geometry (Å, º)

**Table:**

*D*—H···*A*	*D*—H	H···*A*	*D*···*A*	*D*—H···*A*
C3—H3···O4^i^	0.98 (3)	2.46 (3)	3.296 (2)	142.6 (19)
C6—H5···O1^ii^	0.96 (3)	2.51 (3)	3.444 (2)	167 (2)
C11—H11*A*···O2^ii^	1.03 (3)	2.60 (3)	3.245 (2)	120.5 (18)

Symmetry codes: (i) −*x*+1, *y*+1/2, −*z*+3/2; (ii) −*x*+1, −*y*, −*z*+1.
